# Evolution of the tumor immune landscape during treatment with tebentafusp, a T cell receptor-CD3 bispecific

**DOI:** 10.1016/j.xcrm.2025.102076

**Published:** 2025-04-15

**Authors:** Joseph J. Sacco, Peter Kirk, Emma Leach, Alexander N. Shoushtari, Richard D. Carvajal, Camille Britton-Rivet, Sophie Khakoo, Laura Collins, Luis de la Cruz-Merino, Zeynep Eroglu, Alexandra P. Ikeguchi, Paul Nathan, Omid Hamid, Marcus O. Butler, Sarah Stanhope, Koustubh Ranade, Takami Sato

**Affiliations:** 1 Clatterbridge Cancer Center – NHS Foundation Trust, Wirral, UK; 2 University of Liverpool, Liverpool, UK; 3 Immunocore Ltd, Abingdon, UK; 4 Immunocore, Rockville, MD, USA; 5 Memorial Sloan Kettering Cancer Center, New York, NY, USA; 6 Weill Cornell Medical College, New York, NY, USA; 7 Northwell Health Cancer Institute, New Hyde Park, NY, USA; 8 Cold Spring Harbor Laboratory Cancer Center, Cold Spring Harbor, NY, USA; 9 Oncology Department, Virgen Macarena University Hospital, Department of Medicine, School of Medicine, University of Seville, 41009 Seville, Spain; 10 Moffitt Cancer Center, Tampa, FL, USA; 11 Department of Melanoma Medical Oncology, The University of Texas MD Anderson Cancer Center, Houston, TX, USA; 12 Mount Vernon Cancer Centre, Northwood, UK; 13 University College London Hospital, London, UK; 14 The Angeles Clinical and Research Institute, a Cedars-Sinai Affiliate, Los Angeles, CA, USA; 15 Princess Margaret Cancer Centre, Department of Medical Oncology and Hematology, Toronto, ON, Canada; 16 Department of Medicine and Department of Immunology, University of Toronto, Toronto, ON, Canada; 17 Sidney Kimmel Cancer Center, Jefferson University, Philadelphia, PA, USA

**Keywords:** uveal melanoma, T cell engager, bispecific, immunotherapy, gp100, tumor immunology, tumor microenvironment

## Abstract

Metastatic uveal melanoma is an aggressive disease with poor outcome, which is refractory to immune checkpoint inhibitors. A T cell receptor (TCR)-based CD3 bispecific, tebentafusp, delivers clinical benefit in patients with metastatic uveal melanoma. Understanding the molecular basis for the anti-tumor activity of tebentafusp in an indication where checkpoint inhibitors are ineffective could aid in identification of other solid tumor indications where CD3 bispecifics may serve an unmet need. By analyzing tumor biopsies taken prior to treatment, early on-treatment, and at progression (NCT02570308), using RNA sequencing (RNA-seq) and immunohistochemistry (IHC), we show that expression of interferon-related genes in the tumor prior to treatment is associated with improved overall survival and tumor reduction on tebentafusp, that T cell recruitment occurs even in tumors with a low baseline level of T cell infiltration, and that durability of changes induced in the tumor microenvironment is key for survival duration.

## Highlights

• Clinical outcome on tebentafusp is independent of baseline T cell infiltration
• IFN-related gene expression in tumor at baseline is associated with longer OS
• Tebentafusp treatment results in increased T cell infiltration of tumor
• Antigen presentation machinery (APM) expression at progression is associated with longer OS

## Graphical abstract

**Figure:**
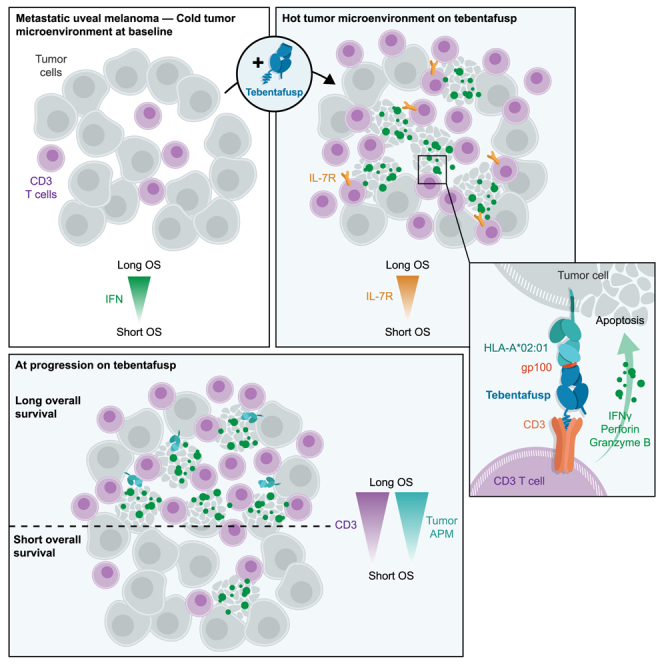

## Teaser

Tebentafusp is a TCR-based CD3 bispecific approved for the treatment of metastatic uveal melanoma in HLA-A∗02:01+ patients. Sacco et al. show how the tumor microenvironment influences response to tebentafusp and how tebentafusp remodels the tumor microenvironment. Implications for combination therapy may apply more broadly to T cell engagers in solid tumor indications.

## Introduction

Uveal melanoma (UM) is a rare tumor type arising in the eye. While treatment of the primary tumor (using radiotherapy or surgery) is almost always successful, in up to 50% of cases systemic metastases develop, most frequently in the liver. In patients with metastatic UM overall survival (OS) is historically around 1 year.

Much of the broad molecular understanding of UM is to date derived from primary tumor samples comprehensively analyzed by The Cancer Genome Atlas Program (TCGA)^1^ as well as earlier work that focused on individual genes and pathways.^2^^,^^3^ The immune landscape of these primary tumors is highly associated with the underlying mutational and chromosomal structural characteristics that dominated patient outcome, specifically the classification of primary tumors based on loss of chromosome 3 and associated alteration in the *BAP1* gene (encoding BRCA1 associated deubiquitinase 1). These genomic factors have been shown across multiple studies to increase the likelihood of metastasis and disease progression.^4^ Loss of chromosome 3/*BAP1* alteration in UM is also associated with increased expression of immune-related genes within the primary tumor.^4^ The strength of this association has led multiple studies to attribute the progression of UM to an inflammatory tumor microenvironment (TME) characterized by T cell and macrophage infiltration and increased expression of human leukocyte antigen (HLA).^5^^,^^6^^,^^7^^,^^8^ This has led to a view of UM as a cancer where the immune infiltrate is a negative prognostic factor, in contrast to what is seen in cutaneous melanoma, although studies on the immune infiltrate in metastatic samples remain limited. The potential for this immune infiltrate to be harnessed by immunotherapies to reverse prognosis in the metastatic setting has not been realized in trials of checkpoint inhibitors (CPIs), which have shown markedly inferior activity in UM compared to cutaneous melanoma.^9^^,^^10^ This may in part be due to the low mutational burden of UM^11^ and the high incidence of hepatic metastasis, both of which are associated with poor response to CPIs.^12^^,^^13^ Several studies have made the point that the failure of CPIs blocking programmed cell death 1 (PD1) and cytotoxic T-lymphocyte associated protein 4 (CTLA4) pathways in metastatic UM may be due to the higher importance of additional inhibitory pathways; while prevalence of expression of PD-L1 (the ligand for PD1) is lower in UM than in cutaneous melanoma,^14^ studies of primary and metastatic UM tumors have found elevated expression of TIGIT, IDO1, TIM3, and LAG3.^15^^,^^16^

Despite multiple studies of immunotherapies and combination therapy in metastatic UM,^17^^,^^18^ only tebentafusp, a T cell receptor (TCR) × CD3 bispecific that targets a specific peptide from the melanoma protein gp100 presented by HLA-A∗02:01, has shown superior OS benefit and is approved for the treatment of metastatic UM.^19^^,^^20^^,^^21^ The success of tebentafusp where CPIs have proved ineffective may be attributed to fundamental differences in mechanism of action. As a first-in-class ImmTAC (immune-mobilizing monoclonal T cell receptor against cancer), tebentafusp binds to target peptide-HLA on the surface of a tumor cell and to CD3 on the surface of a T cell, resulting in immune synapse formation regardless of T cell antigen specificity.^22^^,^^23^ The immune synapse initiates intracellular processes that result in T cell effector function, including induction of target-cell death and interferon gamma (IFNγ) release.^24^ IFNγ in turn induces release of chemokines,^25^ which recruit more T cells to the tumor.^26^ In contrast, the activity of CPIs is dependent on the presence of rare pre-existing tumor-specific T cells to deliver anti-tumor activity, either by rescuing these cells from an exhausted phenotype or by promoting T cell priming and clonal expansion in the lymph node. Both mechanisms may be dependent on high tumor mutational burden and a favorable immune environment, restricting the indications susceptible to checkpoint inhibition.

While gene expression and mutational status of primary UM and their association with disease progression have been extensively studied by TCGA, publicly available data for metastatic UM are more limited.^15^^,^^27^ As part of the IMCgp100-102 clinical trial, we collected comprehensive molecular data from patients being administered tebentafusp in the treatment of metastatic UM and analyzed these datasets to understand how the activity of a CD3 bispecific reshapes the TME. As tebentafusp is a T cell engager, we focused on immune-related genes, and in particular those related to T cell function and phenotype. We looked for gene expression signatures relating to the tebentafusp mechanism of action and for changes that correlated with tumor reduction and OS, to understand why this TCR-CD3 bispecific is effective in an indication where other immunotherapies have failed. Additionally, by analyzing tumor biopsies collected at the time of progression on tebentafusp, we identified a molecular phenotype that corresponds to prolonged survival even after radiographic progression.

## Results

### Immune landscape of metastatic UM

**Figure 1:**
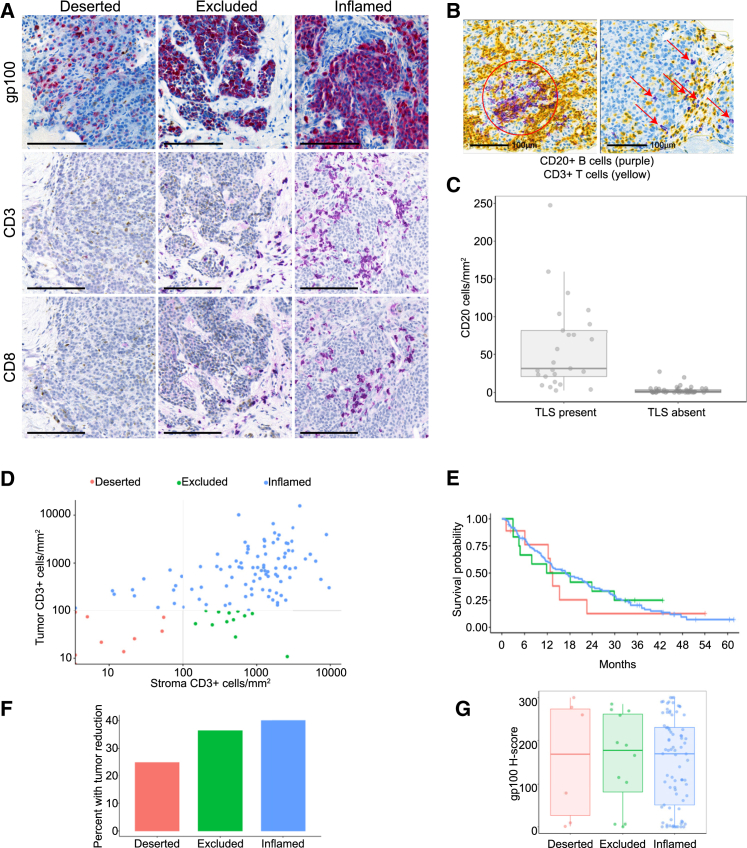
Immune status of tumors prior to treatment with tebentafusp Tumor biopsies collected prior to treatment were analyzed by IHC. (A) Representative images from tumors categorized as deserted, excluded, and inflamed, stained for gp100 (red), CD3 (purple), and CD8 (purple). 100 μm scale bar shown. (B) Representative images of B cells within (circled) and in absence of (arrows) lymphoid aggregates (B cell, CD20, purple; T cell, CD3, yellow). 100 μm scale bar shown. (C) Plot of abundance of CD20^+^ cells in tumor stratified by presence/absence of lymphoid aggregates (21-fold difference, *p* = 1.4E−10). Median and interquartile range are indicated; *n* = 70. (D) Plot of abundance of CD3^+^ cells in tumor and peritumoral stroma, classifying T cell infiltration status as deserted, excluded, or inflamed; *n* = 146. (E) Kaplan-Meier plot of OS of patients stratified by T cell infiltration status at baseline. No significant difference between groups (*p* = 0.904, Cox regression); *n* = 96 inflamed, 12 excluded, 9 deserted. (F) Incidence of tumor reduction (sum of lesion diameters [SLD] at any on-treatment time point below pre-treatment SLD) stratified by T cell infiltration status. No significant difference (*p* = 0.802, Fisher’s exact test); *n* = 85 inflamed, 11 excluded, 8 deserted. (G) Boxplot of gp100 protein level in tumor cells, stratified by T cell infiltration status. Median and interquartile range are indicated.

To explore how the molecular and cellular context of metastatic UM affects response to tebentafusp, we analyzed pre-treatment biopsies from a phase 1/2 clinical study, NCT02570308 (Table 1),^28^^,^^29^ by immunohistochemistry (IHC) and RNA sequencing (RNA-seq). Site of biopsy was liver metastasis for 59 of 71 (83%) baseline biopsies analyzed by RNA-seq and 115 of 147 (78%) baseline biopsies analyzed by IHC (Tables S1, S4, and S8). Selection of a focused set of immune markers for use in this study was informed by the T cell-dependent mechanism of action of tebentafusp and by earlier studies that used a broader panel of immune markers.^30^ We first assessed the abundance of T cells in pre-treatment tumor biopsies by IHC (Figures 1A and S1). Cells positive for pan-T cell marker CD3 were detected in biopsies from 117 of 120 patients, with median CD3^+^ cell density within the tumor 499 per mm^2^ (interquartile range 144–1102). Cells positive for CD8 were detected in biopsies from 115 of 120 patients, with median 223 per mm^2^ (interquartile range 73–584). B cells, a positive prognostic in cutaneous melanoma,^31^ were less abundant, with a median level of 3.23 CD20-positive cells/mm^2^ (interquartile range 0.9–13.5). CD20-positive cells were seen within and outside of lymphoid aggregates (LAs) (Figure 1B) and were significantly more abundant in biopsies where LAs were present (Figure 1C). Presence of LAs at baseline was not associated with outcome on tebentafusp.

**Table 1:** Patient characteristics

Number of patients	146
Age, median (range), years	61 (25–88)
Male sex, *n* (%)	72 (49)
ECOG status, *n* (%)	
0	103 (71)
1	43 (29)
Lactate dehydrogenase > Upper limit of normal, *n* (%)	85 (58)
Alkaline phosphatase > Upper limit of normal, *n* (%)	47 (32)
Time from primary diagnosis to metastatic disease, median (range), years	3 (0–28)
Number of prior anti-cancer therapy regimens in metastatic setting, *n* (%)	
0 prior lines	3 (2)
1 prior line	89 (61)
2+ prior lines	54 (37)
Previous anti-cancer therapy type in metastatic setting, *n* (%)	
Anti-PD1/anti-PD-L1 monotherapy	57 (39)
Anti-CTLA4 monotherapy	13 (9)
Anti-CTLA4 and anti-PD1	34 (23)
UM driver mutations detected in 63 tumor biopsies, *n* (%)	
*GNAQ*	26 (41)
*GNA11*	23 (37)
*PLCB4*	1 (2)
*CYSLTR2*	3 (5)
*SF3B1*	11 (17)
*BAP1*	3 (5)
*BAP1* copy-number alterations in 63 tumor biopsies	*n* (%)
Gain of at least one copy	1 (2)
Diploid	30 (48)
Loss of one copy	32 (51)

The immunological phenotype of tumors can be classified on the basis of abundance of immune cells within tumor tissue and peritumoral stroma.^32^ We found that the majority of tumor biopsies contained significant numbers of T cells within the tumor tissue, an “inflamed” phenotype (Figure 1D), with a small proportion largely devoid of T cells within the tumor, and either containing T cells within the peritumoral stroma (“immune-excluded”) or lacking T cells in both regions (“immune-deserted”). There was no significant difference in OS or in the proportion of patients with any degree of tumor reduction between these categories (Figures 1E and 1F). This contrasts with CPIs, which deliver less benefit in patients with immune-deserted tumors.^33^ Tebentafusp targets a peptide derived from the protein gp100, when presented by HLA-A∗02:01. Analysis of gp100 protein expression by IHC revealed a broad range of expression levels in tumors from all three immune infiltration categories (Figure 1G). Mutations in *GNAQ* and *GNA11* were found in 37% and 41% of patients, respectively, with mutations in *SF3B1* in 17% and *BAP1* mutation or loss in 51% (Figure S2). There was no significant difference in the frequency of UM driver mutations (*GNAQ*, *GNA11*, *SF3B1*, and *BAP1*) or *BAP1* copy number variation (CNV) between the three T cell infiltration categories, and no significant association of mutation status with OS.

### High expression of IFN pathway genes identifies a subset of patients with metastatic UM with better outcomes on tebentafusp

To identify pathways associated with survival on tebentafusp, we analyzed gene expression in pre-treatment biopsies by RNA-seq (Table S5). Whole transcriptome correlation analysis indicates three distinct clusters (Figure S3). The genes with greatest differential expression between clusters are strongly enriched for normal hepatic function genes including *CES5A*, *SLC22A1*, and *CYP1A2*, suggesting that the level of hepatocyte content is a major driver of the clustering. This is confirmed by gene set enrichment analysis (GSEA) analysis for liver-specific genes. Differential gene expression analysis between biopsies from liver vs. other sites showed an elevated level of hepatocyte-specific genes and an enrichment of associated metabolic pathways including mono-oxygenase and oxidoreductase (Figure S4). Melanoma-specific genes *PMEL* and *PRAME* and outcome-associated genes discussed in the following paragraph did not show any significant differences in expression (*p* < 0.01) in tumor biopsies from liver vs. non-liver sites.

**Figure 2:**
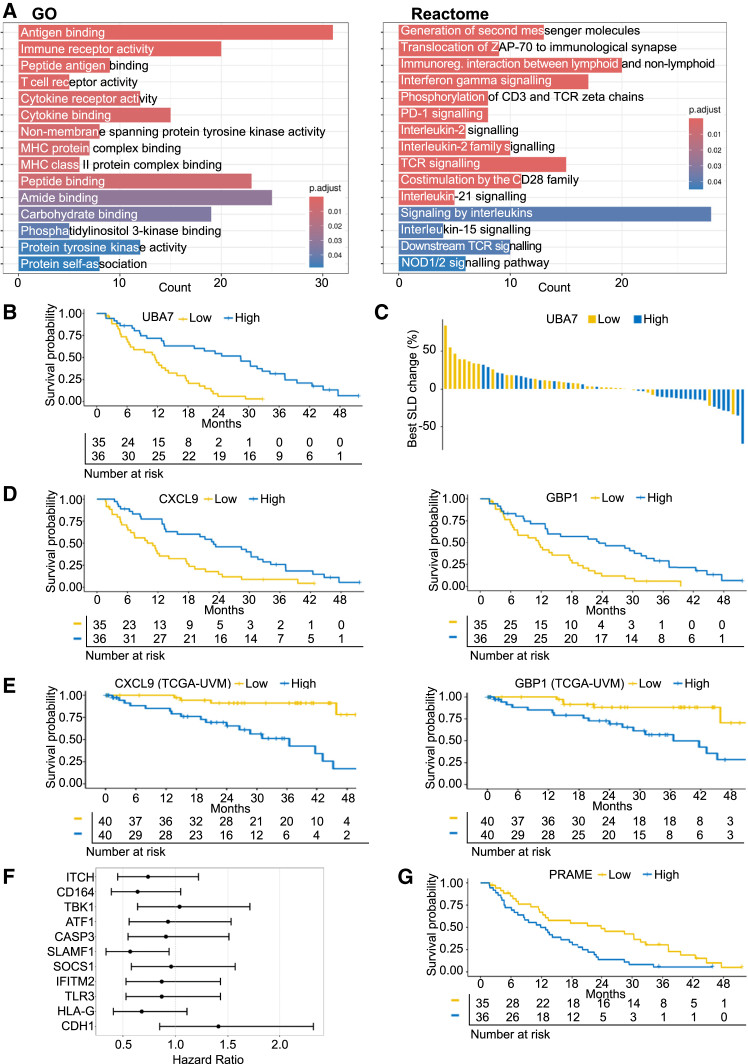
Baseline expression of genes related to antigen presentation and interferon signaling is associated with outcome Gene expression in baseline tumor biopsies (*n* = 71) was analyzed by RNA-seq. (A) Gene Ontology and Reactome pathway analysis of genes associated with longer OS. (B and C) Kaplan-Meier plot of OS (HR = 0.3 [95% CI 0.18–0.56], *p* < 0.001) and (C) waterfall plot showing best reduction in SLD (OR = 0.13 [95% CI 0.03–0.47], *p* = 0.001), both stratified at median tumor *UBA7* expression at baseline. (D) Kaplan-Meier plots of OS stratified by median expression in baseline tumor biopsy of *CXCL9* (HR = 0.45 [95% CI 0.27–0.75], *p* = 0.002) and *GBP1* (HR = 0.42 [95% CI 0.24–0.71], *p* = 0.001). (E) Kaplan-Meier plots of OS data from TCGA-UVM, stratified by median expression of *CXCL9* (HR = 5.85 [95% CI 2.15–15.91], *p* < 0.0001) and *GBP1* (HR = 4.38 [95% CI 1.62–11.82], *p* = 0.001). (F) Forest plot showing OS HR values and 95% confidence intervals for a set of genes reported to be associated with improved survival on CPIs. *HLA-DRB4* not shown as expression level was below threshold for inclusion. (G) Kaplan-Meier plot of OS stratified by median *PRAME* gene expression in baseline tumor biopsy (HR = 2.0 [95% CI 1.2–3.4], *p* = 0.007).

We assessed all genes for association with OS on tebentafusp and performed enrichment analysis using Gene Ontology (GO) and Reactome genesets (Figure 2A). GO terms significantly enriched in survival-associated genes included multiple terms relating to antigen presentation and cytokine receptor activity. Consistent with the mechanism of tebentafusp as a T cell activator, multiple reactome pathways involved in T cell activation showed an association with survival, as well as pathways associated with IFNγ and interleukin (IL)-2 family signaling. Individual genes significantly associated (*p* < 0.01) with both tumor reduction and OS are shown in Table S2. Of the 38 genes identified, the gene with the strongest association with outcome (using a combined readout of tumor reduction and OS) was *UBA7*, which encodes an E1-like ubiquitin-activating enzyme involved in conjugation of *ISG15* to proteins (ISGylation), a key process in the response to type-I IFN.^34^^,^^35^^,^^36^ Patients with above-median expression of *UBA7* prior to treatment had longer OS than patients with below-median expression (hazard ratio [HR] = 0.31 [95% confidence interval (CI) 0.18–0.56], *p* < 0.0001; Figure 2B). Tumor reduction was observed in 19 of 32 (59%) patients with above-median *UBA7* expression, compared with just 5 of 32 (16%) patients with below-median *UBA7* expression (odds ratio [OR] = 0.13 [95% CI 0.03–0.47], *p* = 0.001; Figure 2C). Given the significant enrichment of Reactome IFNγ signaling pathway among OS-associated genes, and role of *UBA7* in IFN signaling, we screened the list of genes significantly associated with OS for additional IFN-stimulated genes (ISGs) and identified ISGs preferentially induced by type I IFN^37^ (e.g., *EPSTI1,* HR = 0.48 [95% CI 0.29–0.81], *p* = 0.005), and those preferentially induced by type II IFN^37^ (e.g., *CXCL9,* HR = 0.45 [95% CI 0.27–0.75], *p* = 0.002). These results indicate that IFN signaling in the tumor at baseline is associated with enhanced anti-tumor activity of tebentafusp.

To assess the relative predictive and prognostic characteristics of IFN pathway expression, we tested whether the genes identified in our analysis are also associated with outcome in the TCGA-UVM dataset (with the caveat that TCGA-UVM is based on primary UM). Interestingly, we found a number of genes that showed reversed association with survival between our dataset and the TCGA primary UM dataset. Looking only at genes with a significant association with outcome in both datasets (*p* < 0.05), there were 19 genes negatively associated with OS in the TCGA dataset but positively associated with OS in our dataset (*UBA7* expression was not associated with outcome in TCGA: HR = 0.91 [95% CI 0.4–2.06]). This set is enriched for immune-related genes including *TESPA1*, *IL2RB*, *LTB*, *CD247* (*CD3z*), and *CXCL9*. Kaplan-Meier plots are shown for two examples, *CXCL9* and *GBP1* (Figure 2D); above-median expression of each gene is associated with improved survival on tebentafusp (HR values of 0.45 [95% CI 0.27–0.75], *p* = 0.002, and 0.42 [95% CI 0.24–0.71], *p* = 0.001, respectively). In contrast, above-median expression of these genes is associated with worse survival in the TCGA dataset in which no patient was treated with tebentafusp (HR values of 5.9 [95% CI 2.2–15.9], *p* < 0.0001, and 4.4 [95% CI 1.6–11.8], *p* = 0.001, respectively, Figure 2E). This crossover of immune-related genes from a negative association with outcome in TCGA to a positive one on tebentafusp treatment is consistent with both the view of immune infiltration being a negative prognostic in primary UM^38^ and the T cell re-directing mechanism of action of tebentafusp.

A previous immune-profiling study of patients with UM treated with immunotherapy, including both primary and metastatic disease, identified a 12-gene signature upregulated at baseline in responders vs. non-responders to subsequent CPI treatment.^39^ Querying this gene set (*CDH1*, *HLA-DRB4*, *HLA-G*, *TLR3*, *IFITM2*, *SOCS1*, *SLAMF1*, *CASP3*, *ATF1*, *TBK1*, *CD164*, and *ITCH*) against our data (Figure 2F), only *SLAMF1* was significantly associated with OS on tebentafusp (HR = 0.57, 95% CI 0.34–0.94, *p* = 0.026), suggesting a difference in the molecular phenotypes favoring response to tebentafusp and CPIs. In the PEMDAC trial in metastatic UM, CCL21 levels in blood were found to be associated with outcome on CPI therapy.^40^ We did not assess CCL21 levels in blood, but patients in the upper quartile for *CCL21* gene expression in tumor had longer OS (HR = 0.43, 95% CI 0.23–0.79, *p* = 0.005), suggesting that this biomarker may be prognostic.

In addition to immune-related genes, we examined the association between the expression of tumor-specific genes and outcomes on tebentafusp. *PRAME* gene expression has been identified as a marker of poor prognosis in UM, associated with an increased risk of metastasis independent of UM driver mutations,^41^ and is associated with 8q amplification.^42^^,^^43^ In our metastatic UM population, above-median *PRAME* expression was associated with shorter OS (Figure 2G). Amplification of 8q was seen in 50 of 63 patients and was associated with higher PRAME expression (Figure S5). There were no significant gender differences in the association of UBA7, CXCL9, and GBP1 with longer OS and PRAME expression with shorter OS.

### Tebentafusp drives significant immune infiltration even in tumors with low T cell infiltration at baseline

**Figure 3:**
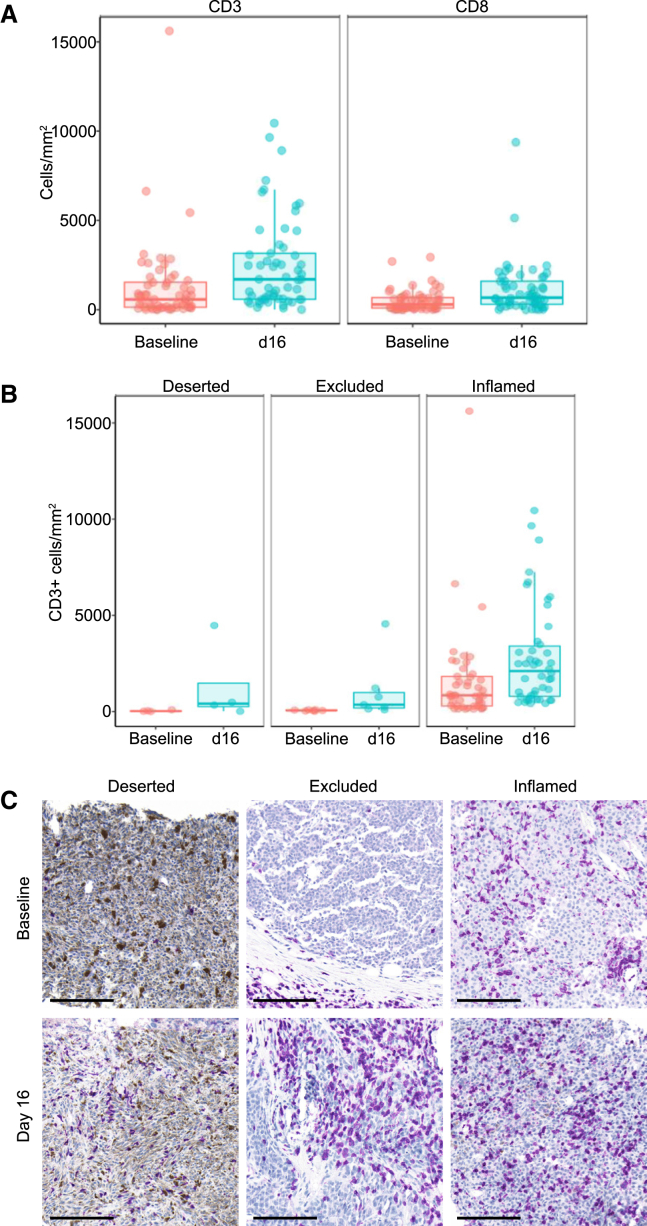
Tebentafusp induces significant tumor immune infiltration even in tumors with low initial T cell infiltration Paired tumor biopsies taken at baseline and day 16 of tebentafusp treatment were analyzed by immunohistochemistry for CD3 (*n* = 57 pairs) and CD8 (*n* = 56 pairs). (A) Boxplot showing abundance of CD3^+^ and CD8^+^ cells in tumor regions. Median and interquartile range are indicated. Increase at day 16 2.9-fold for CD3 (*p* < 0.0001) and 2.3-fold for CD8 (*p* < 0.0001). (B) Boxplot showing abundance of CD3^+^ cells in tumor by baseline T cell infiltration status (see Figure 1B). Median and interquartile range are indicated. Increase at day 16 16.9-fold for deserted (ns, *p* = 0.25), 6.8 for excluded (*p* = 0.016), and 2.5-fold for inflamed (*p* < 0.001). (C) Representative images showing CD3 (purple) at baseline and day 16, from patients with deserted, excluded, and inflamed tumors. Brown pigmentation is melanin. 50 μm scale bar shown.

To assess how tebentafusp reshapes the tumor immune microenvironment, additional biopsies were collected at day 16, the day after the third dose of tebentafusp (Table S1). Due to the step-up dosing regimen used, this third dose was the first at the target dose level of 68 μg. Analysis of paired biopsies by IHC (*n* = 57 and *n* = 56 pairs for CD3 and CD8 stain, respectively) revealed a substantial 2.9-fold increase (*p* < 0.0001) in median T cell infiltration and a 2.3-fold increase (*p* < 0.0001) in median CD8^+^ cell numbers at day 16 (Figures 3A and S6A). Significant increases in CD3^+^ and CD8^+^ cell infiltration at day 16 were observed in both male and female patients.

Stratifying by baseline T cell infiltration status as defined earlier, we find numerical increases in T cell frequency on-treatment in immune-deserted, immune-excluded, and inflamed tumors (Figures 3B, S6B, and 3C). Notably, the immune-deserted subset showed the greatest median increase (17-fold). This demonstrates the ability of tebentafusp treatment to drive T cell recruitment even in this tumor category that is regarded as particularly resistant to CPI therapy.^32^^,^^44^ Immune-excluded and inflamed subsets also demonstrated substantial increases in T cell infiltration on tebentafusp treatment (6.8-fold and 2.7-fold, respectively; *p* = 0.016 and *p* < 0.001).

### Tebentafusp induces high levels of T cell activation and counter-regulatory signaling in metastatic tumors after three doses

**Figure 4:**
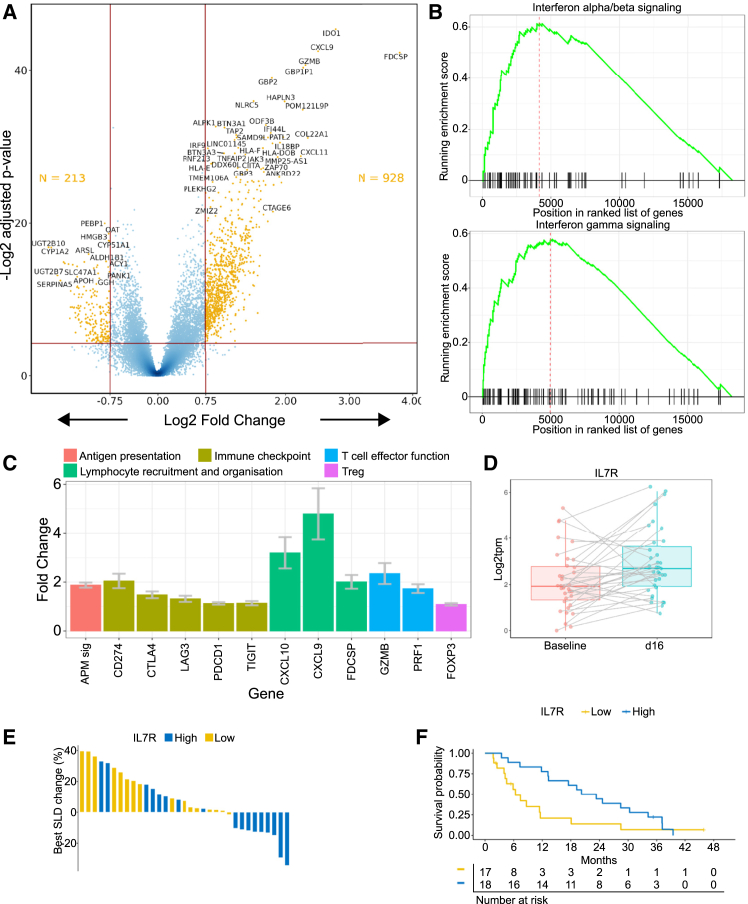
Tebentafusp induces T cell activation at day 16 Tumor biopsies taken at day 16 of treatment were analyzed for gene expression by RNA-seq. Differential gene expression analysis was performed against paired baseline biopsies (*n* = 35 pairs). (A) Volcano plot indicating change in gene expression in tumor from baseline to day 16. Differentially expressed genes are shown in yellow. (B) GSEA plot of genes ranked by increase in expression in between baseline and day 16, using interferon alpha/beta signaling and interferon gamma signaling gene sets. (C) Fold change in expression of selected immune-related genes between baseline and day 16. Data are represented as median and standard error. (D) Boxplot of *IL7R* expression in tumor biopsy at baseline and day 16 (1.7-fold increase, *p* = 0.003). Median and interquartile range are indicated. Lines join paired biopsies. (E) Waterfall plot showing maximum tumor reduction stratified at median of expression of *IL7R* in tumor at day 16 (OR = 0.06 [95% CI 0–0.61], *p* = 0.007). (F) Kaplan-Meier plots of OS stratified at median of expression of *IL7R* in tumor at day 16 (HR = 0.47 [95% CI 0.23–0.98], *p* = 0.04).

To understand the functional status of the infiltrating lymphocytes, we analyzed gene expression in day 16 tumor biopsies, collected approximately 24 h after the third dose of tebentafusp (Table S6). Differential gene expression analysis between tumor samples at baseline and day 16 revealed substantial changes to gene expression (Figure 4A). The most differentially expressed genes were *IDO1*, *CXCL9*, and the follicular dendritic cell-associated gene *FDCSP*, which were upregulated 7-fold, 6-fold, and 14-fold on-treatment, respectively, with significant upregulation in both male and female patients. Applying GSEA to the differentially expressed genes revealed a significant enrichment of both IFNα/β-related genes and IFNγ-related genes among genes upregulated on-treatment (Figure 4B).

Well-characterized ISGs with significant upregulation at day 16 included *CXCL9*, *CXCL11*, *ETV7*, *GBP2*, *IFI44L*, and *GBP1* (ranging from 8.7-fold to 4.5-fold increase, *p* < 0.001). *UBA7*, discussed earlier in the context of association of baseline expression with outcome, was also significantly upregulated (2-fold, *p* = 0.0001) on-treatment. This upregulation on-treatment of genes associated at baseline with improved outcome suggests that tebentafusp modifies the TME in a way that promotes further anti-tumor activity.

Targeted interrogation of the differential expression data identified significant increases in expression of genes indicative of T cell effector functions including cytotoxicity (*GZMB*, increased 2.5-fold; *PRF1*, increased 2.4-fold) and genes associated with lymphocyte recruitment and organization (*CXCL9*, increased 8.7-fold; *CXCL10*, increased 3.2-fold; *FDCSP*, increased 5.1-fold) (Figure 4C). As tebentafusp targets peptide presented in the context of class I HLA, it is noteworthy that expression of an antigen presentation pathway gene signature (*B2M*, *HLA-A*, *TAP1*, *TAP2*, *TAPBP*, *PSMB8*, and *PSMB9*) increased on-treatment (2-fold change, *p* = 0.0019). Induction of immune checkpoint genes was marginal (*PDCD1* [PD1], increased 1.2-fold; *LAG3*, increased 1.2-fold; *TIGIT*, increased 1.3-fold). Consistent with strong T cell activation and IFN pathway induction, *CD274* (PD-L1) and *CTLA4* expression was increased almost 2-fold on tebentafusp. *FOXP3* expression was low and was not induced on-treatment suggesting an absence of regulatory T cell recruitment.

Inference of natural killer (NK) cell abundance from bulk transcriptome data is complicated by T cell expression of many genes used to define NK cell signatures. Genes with the greatest enrichment in NK cells relative to T cells, *KLRB1* and *KLRF1*,^45^ did not show significant upregulation, suggesting that NK cells are not a major component of the lymphocytes recruited in metastatic UM. Similarly, genes characteristic of myeloid-derived suppressor cells (*ARG1* and *NOS2*) were not increased on-treatment.

Of particular note was the induction of genes involved in pyroptosis/necroptosis cell death pathways, including inflammasome components *NLRP3*, *PYCARD* (encoding ASC protein), and *CASP1* and cell death effectors (*RIPK3*, *MLKL*, *GSDMB*, and *GSDMD*) (Table S3). In contrast to apoptosis, necroptosis and pyroptosis are proinflammatory and are associated with neoantigen release and maturation of professional antigen-presenting cells,^46^ which may promote an endogenous anti-tumor immune response.

### Naive/memory-like T cells recruited to tumors may be the principal drivers of anti-tumor immunity on tebentafusp

We next investigated the association of relevant T cell subsets in the tumor on-treatment with outcome. Expression of broadly expressed T cell markers such as *CD3D*, *CD3E*, and *CD3G* at day 16 of treatment, i.e., after 3 doses of tebentafusp, was increased but was not associated with tumor reduction or OS (data not shown). By contrast, expression of *IL7R*, a marker of naive/memory-like cells absent from most late-activated and exhausted T cells,^47^ was increased at day 16 (1.7-fold, *p* = 0.003) compared to baseline (Figure 4D), and *IL7R* expression at day 16 was strongly associated with tumor reduction (OR = 0.06 [95% CI 0–0.61], *p* = 0.007) (Figure 4E). Patients with above-median IL7R expression in tumor at day 16 also showed improved OS (HR = 0.47 [95% CI 0.23–0.98], *p* = 0.04, Figure 4F). As IL7R expression by naive T cells is downregulated following T cell activation through the TCR/CD3 complex,^48^ *IL7R* expression in the tumor at day 16 may reflect the number of recently recruited naive T cells. In line with this hypothesis, expression of *IL7R* at baseline was not associated with tumor reduction. The association of *IL7R* expression after 3 doses of tebentafusp (but not prior to initiation of tebentafusp) with tumor reduction and OS suggests that recently recruited naive cells are key mediators of the anti-tumor activity of tebentafusp. While *IL7R* expression is associated with naive/central memory T cells, its expression is not exclusive to this subset and is also seen on some late-activated and effector memory populations.^47^ In light of this, we assessed additional markers associated with naive/memory-like T cells (*CCR7*, *TCF7*, and *LEF1*) and found them to be highly correlated with *IL7R* expression (Spearman’s R = 0.78, 0.69, and 0.68, respectively, Figure S7).

### High target expression level is associated with greater T cell infiltration and activation on-treatment

**Figure 5:**
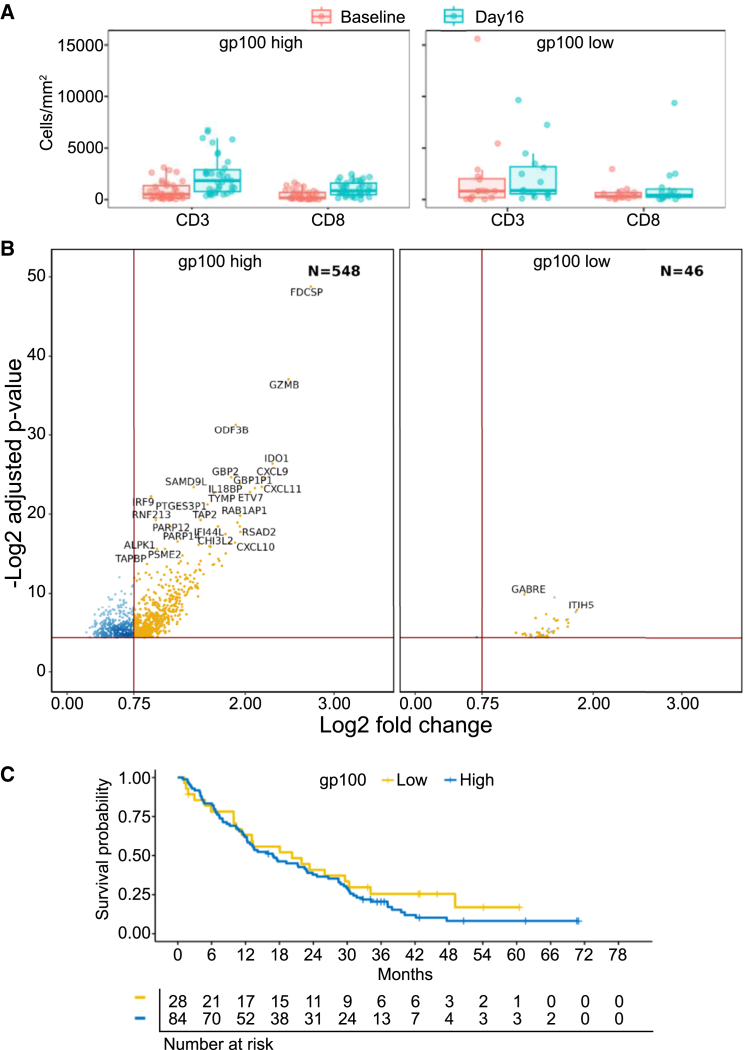
Target expression is associated with greater T cell infiltration and activation on tebentafusp (A) Box plot of number of cells in tumor regions of paired biopsies expressing CD3 and CD8 (by IHC) at baseline and at day 16, stratified at lower quartile of baseline gp100 expression. Median and interquartile range are indicated. *n* = 50 pairs. (B) Volcano plots showing genes upregulated at day 16 compared to baseline in paired biopsies, stratified at lower quartile of baseline gp100 expression. *n* = 35 pairs. (C) Kaplan-Meier plot of OS stratified at lower quartile of baseline gp100 expression (HR = 0.79 [95% CI 0.49–1.29], not significant; *p* = 0.35).

Metastatic UM tumors show a wide range of expression levels of gp100, the target of tebentafusp. We assessed the impact of gp100 expression level on tebentafusp-induced changes to immune infiltration and gene expression in the tumor. As ImmTAC molecules are highly sensitive T cell activators, requiring as few as 2–10 target molecules per cell,^23^ we stratified patients at a low target expression threshold: the lower quartile of gp100 IHC H-score. The on-treatment increase in the number of CD3 and CD8 cells was higher in the gp100-high group than in the gp100-low group (3.7-fold vs. 1.1-fold for CD3 and 5.2-fold vs. 1.4-fold for CD8) (Figures 5A and S6C). In comparative analysis of transcriptomic data, tumors with gp100 expression above the lower quartile had a greater number of upregulated genes at day 16 (548, vs. 46 in the low gp100 group), and larger fold changes on-treatment (Figure 5B), with notable differences in upregulation of key immune-related genes including *GZMB*, *IDO1*, and *CXCL9* (upregulated 5.6-fold, 4.9-fold, and 4.7-fold, respectively, in patients with high gp100, vs. non-significant changes of 1.4-fold, 1.7-fold, and 1.5-fold in patients with low gp100). Despite these differences in early on-treatment gene expression changes, OS on tebentafusp was similar across the range of gp100 expression levels (Figure 5C).

### Higher expression of antigen presentation genes in tumor at radiographic progression is associated with improved survival

**Figure 6:**
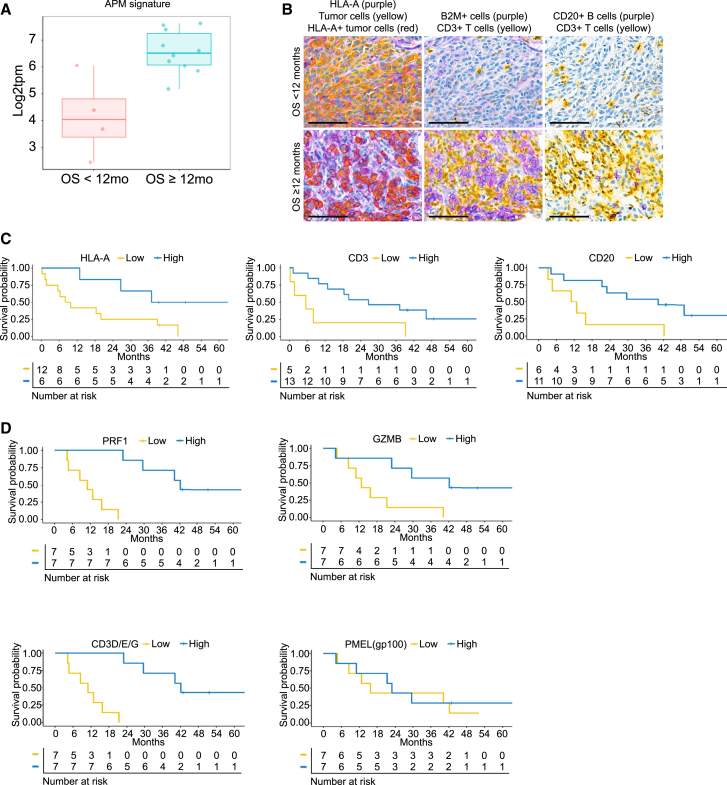
At progression, higher expression of antigen presentation genes and T cell infiltration are associated with longer survival Tumor biopsies collected after radiological progression were analyzed by IHC (*N* = 18, except for CD20 where *n* = 17) and RNA-seq (*n* = 14). (A) Box plot of expression of APM gene signature in tumor biopsies at progression, stratified by duration of OS (5.6-fold higher in long OS, *p* = 0.014). Median and interquartile range are indicated. (B) Representative images of progression biopsies from short OS and long OS patients, stained for (left) HLA-A (purple) and melanoma triple (gp100, MART1, tyrosinase) (yellow), (middle) B2M (purple) and CD3 (yellow), and (right) CD20 (purple) and CD3 (yellow). 100 μm scale bar shown. (C) Kaplan-Meier plots of OS stratified by tumor cell membrane HLA-A staining (HR = 0.3, *p* = 0.038, 95% CI 0.075–1), by CD3 IHC (HR = 0.29, *p* = 0.025, 95% CI 0.09–0.91), and by CD20 IHC (HR = 0.26, *p* = 0.018, 95% CI 0.08–0.85). (D) Kaplan-Meier plot of OS stratified at median of *PRF1* gene expression (HR = 0, *p* < 0.001, 95% CI undefined), *GZMB1* gene expression (HR = 0.194, *p* = 0.015, 95% CI 0.05–0.81), mean expression of *CD3D*, *CD3E*, and *CD3G* (HR = 0, *p* < 0.001, 95% CI NA), and *PMEL* (gp100) gene expression (HR = 0.78, *p* = 0.69, 95% CI 0.24–2.6).

After analyzing the tumor and its microenvironment at baseline and after 3 doses of tebentafusp, we examined how the TME had evolved by the time of radiographic progression on tebentafusp. As tebentafusp treatment confers an OS benefit even among patients with radiographic progression,^49^ many patients in this study population were treated beyond progression and survived for an extended period beyond progression. Optional biopsies were collected from 18 patients after progression according to Response Evaluation Criteria in Solid Tumors (RECIST). Median time for collection of progression biopsies was 5.4 months from initiation of treatment with tebentafusp (Figure S8). These were analyzed by IHC and RNA-seq (Table S7) to identify factors present late on-treatment that are associated with duration of survival. Gene expression analysis showed 6-fold higher expression of an antigen presentation machinery (APM) gene signature at progression in patients with long OS (≥12 months from start of treatment) compared to those with shorter OS (<12 months, Figure 6A), with expression of all component genes significantly higher (2.3-fold–7.6-fold). We confirmed this result for HLA-A by devising a double staining method that enabled determination of levels of HLA-A on tumor cell membranes. Patients with higher expression of membrane HLA-A on tumor cells at progression showed significantly longer OS (HR = 0.3 [95% CI 0.075–1]) (Figures 6B and 6C). Higher T cell (CD3^+^) infiltration of tumors was also associated with longer survival (HR = 0.29 [95% CI 0.09–0.91]), as was the level of B cell (CD20^+^) infiltration (HR = 0.26 [95% CI 0.08–0.85]).

Consistent with the IHC data, patients with higher tumor expression of *CD3D/E/G* genes at progression showed significantly longer survival; median OS was 42 months for above-median vs. 11 months in the below-median group (Figure 6D). Above-median expression of *GZMB* and *PRF1* in tumor at progression was also associated with longer median OS (42 months vs. 13 months and 42 months vs. 11 months, respectively). These observations suggest that persistence of antigen presentation within the tumor leads to continued maintenance and activation of T cells, resulting in long OS. In contrast, expression of gp100 at progression did not show an association with survival, suggesting that downregulation of target expression is not a significant resistance mechanism.

## Discussion

Tebentafusp delivers remarkable survival benefit, with OS in phase 1/2 trials nearly double the historical benchmark values,^28^^,^^29^ and significantly longer OS than investigator’s choice in a randomized controlled phase 3 trial.^20^^,^^21^ Anti-tumor activity is demonstrated by reductions in tumor size and by reductions in circulating tumor DNA, which showed better association with OS than RECIST response. Using a large set of biopsies collected from a phase 1/2 trial of tebentafusp in patients with metastatic UM, we have examined how a solid tumor and its immune microenvironment evolve on T cell engager therapy. Prior to tebentafusp treatment, the majority of tumors were extensively infiltrated by T cells, consistent with previous literature suggesting a higher level of immune infiltration in UM metastases than in primary tumor.^39^ This may be in part due to the enrichment in metastatic patients of monosomy 3, which is associated with a higher rate of metastasis and increased immune infiltration.^1^ Despite this T cell infiltration, metastatic UM is resistant to CPI therapy, which may be due to low tumor mutational burden,^11^ immunosuppressive microenvironment, or T cell dysfunction from chronic antigen exposure.^50^^,^^51^

Increased expression of a number of genes in the IFN pathway was associated with longer survival in this analysis yet showed no significant association or even a negative association with survival in the TCGA-UVM dataset. This difference may in part be explained by the fact that the TCGA-UVM dataset is derived from primary tumors, rather than metastatic disease. In the TCGA-UVM dataset, immune infiltrate and IFN-related gene expression are associated with class II UM tumors (with monosomy of chromosome 3),^1^ which have an increased frequency of metastasis and hence a poor prognosis. The positive association of these genes with outcome on tebentafusp may reflect how the tumor immune infiltrate is harnessed and re-directed by tebentafusp to derive benefit for patients. Thus, the low-level inflammatory environment that may drive progression in the primary setting may promote anti-tumor activity in metastatic patients when treated with tebentafusp.

Tebentafusp-mediated activation of T cells in the presence of target results in secretion of cytokines including IFNγ. This leads to the induction of IFNγ-inducible chemokines (*CXCL9*, *CXCL10*, and *CXCL11*), and within 24 h a mobilization of T cells from the circulation, leading to increased levels of T cells in the tumor by day 16.^30^ In this study, we demonstrate that this increase in T cell infiltration at day 16 is observed in tumors with inflamed, immune-excluded, and immune-deserted phenotypes at baseline, with the greatest fold change in T cell infiltration level seen in patients with immune-deserted tumors. This normalization of T cell infiltration levels on-treatment may explain the similar OS seen in patients across these categories.

The extent of T cell infiltration early on-treatment is dependent on gp100 expression level, with greater T cell infiltration occurring in tumors with higher levels of gp100. This difference in early intratumoral response is not reflected in OS, which showed no association with gp100 level. The association of baseline IFN signature with outcome, and the induction of ISGs (including antigen presentation machinery genes) upon tebentafusp treatment, suggests a positive feedback loop that may explain this apparent paradox: in tumors with low gp100 expression, initial tebentafusp-redirected T cell activation may be insufficient to achieve strong T cell recruitment, but local IFN production leads to increased antigen presentation, leading to a stronger response to subsequent doses; thus, T cell infiltration occurs early in tumors with a higher level of gp100 expression but later in tumors with low gp100. Biopsies taken at day 16 may therefore be too early to see the peak of T cell infiltration for low-gp100 tumors. An additional hypothesis is that tebentafusp induces an endogenous T cell response to other tumor antigens or epitope spread, thereby breaking the association between gp100 expression and long-term outcomes.

An unexpected finding of this study was that genes expressed in naive/memory-like T cells and associated with stemness (such as *IL7R*^52^ and *TESPA1*^53^) were particularly strongly associated with outcome. This may reflect the greater proliferative and self-renewal capacity of naive and stem cell memory T cells.^54^ High intratumoral expression of these genes may also reflect a greater recent influx of T cells from the periphery. T cells recently recruited to the tumor will not have entered an exhausted/dysfunctional state typically found in T cells following sustained exposure to the TME, and so may exhibit greater potential for CD3-stimulated proliferation and activity. Intratumoral IL7R is a positive prognostic for survival in metastatic melanoma,^51^ and in a melanoma model an IL7R-high CD8^+^ T cell population with central memory-like features and lack of exhaustion markers demonstrated superior antitumor activity.^55^ By promoting this T cell gene expression profile in the tumor, tebentafusp may be modifying the TME into a more favorable anti-tumor state.

As noted earlier, in addition to the direct anti-tumor activity of redirected T cells, CD3 bispecific therapy may drive a second mechanism of action, through promoting epitope spread. Early on-treatment, there was an increase in intratumoral expression of *GSDMD*, *RIPK3*, and *MLKL*, genes associated with pyroptosis/necroptosis,^56^ forms of inflammatory cell death that drive release of antigens from tumor cells as well as release of inflammatory factors such as IL-1β and IL-18,^56^ leading to potentiation of anti-tumor immunity.^46^ The recruitment of naive T cells to the tumor, suggested by increased expression at day 16 of *IL7R*, may increase the likelihood of epitope spread, by exposing T cells with a wider range of TCR specificities to tumor antigens.

Although the level of T cell infiltration at day 16 was not associated with outcome, at later on-treatment time points, as shown by biopsies collected at time of radiographic progression, frequency of T cells was strongly associated with duration of survival. Similarly, at these later time points, patients showing long survival had elevated expression of genes associated with antigen presentation, likely reflecting sustained IFN signaling. This suggests that rather than the strength of the early response, it is the ability to sustain antigen presentation and T cell recruitment to, and activation within, the tumor that contributes to longer survival. A similar pattern, with intratumoral immune gene activation at early time points being widespread and lacking an association with outcome, but with later time points showing an association with outcome, has been reported for other immunotherapeutic approaches.^57^

The importance of continued expression of APM genes for prolonged survival is in keeping with the finding that, across a broad range of cancer types, genetic defects in APM, such as loss of heterozygosity of HLA-I and inactivation of B2M, are common mechanisms of immune escape.^58^ One confounding factor in the analysis of optional progression biopsies is that the biopsies from patients with shorter OS (<12 months) were collected earlier than the biopsies from patients with longer OS (median collection time point 2.2 months for short OS, 9 months for long OS). Thus, the phenotype observed in the biopsies from short OS patients may also reflect tumors with more rapidly progressing disease.

The differing sets of genes identified as associated with survival at baseline in this study and in an immune-profiling study of patients with UM treated with immunotherapy^44^ may reflect a key difference in mechanism of action of tebentafusp compared to CPIs; tebentafusp recruits T cells from the blood to the tumor and redirects them to kill tumor cells regardless of their TCR specificity, while CPIs depend on the presence of antigen-specific T cells within the tumor or in the draining lymph node. As a result, T cell dysfunction within the tumor, reflected by epigenetic changes that are not reversed by CPI treatment,^51^^,^^59^ may present a greater obstacle for CPIs than for tebentafusp, as there is an abundant supply of fresh non-exhausted T cells in the periphery that can be recruited to the tumor.

The changes to the tumor immune environment resulting from tebentafusp treatment suggest mechanisms that may promote activity of combination therapies. The tebentafusp-induced increase in APM gene expression, increased inflammatory signal, and recruitment of greater T cell diversity present a rationale for combination with a CPI to promote epitope spread and limit exhaustion of newly expanded tumor-specific T cells. In contrast, there is no association between prior CPI treatment and survival on tebentafusp.^29^^,^^60^ The increase in IFNγ signaling and antigen presentation after tebentafusp treatment may also enhance the response to a second TCR-CD3 T cell engager targeting a different tumor antigen, through increased T cell responsiveness and increased target presentation.

In summary, this study describes the evolution of the tumor immune microenvironment in response to a T cell-engaging bispecific. We identified patterns of gene expression in metastatic UM associated with outcome and changes in immune infiltration and gene expression occurring on-treatment. This dataset is consistent with our understanding of the importance of T cell recruitment and antigen presentation in the response to tebentafusp, and the role of IFN production in remodeling the tumor immune microenvironment to facilitate both. While the present dataset derives entirely from metastatic UM, ImmTAC activity has been demonstrated against multiple targets^61^^,^^62^ and in multiple cancer indications.^63^ With the shared mechanism of action of ImmTAC molecules and of T cell-engaging bispecifics more generally, the molecular predictors of outcome identified here may be of more general relevance.

### Limitations of the study

A limitation common with the use of bulk RNA-seq is that the cellular source of a given gene expression signal is not always clear. Some differences in gene expression between biopsies will be due to varying levels of tumor content and the nature of the surrounding non-tumor tissue. In this clinical trial, we did not have a control arm, so we cannot be certain whether genes associated with outcome are predictive or prognostic. On-treatment gene expression changes at day 16 (24 h post dose) may be dominated by the effects of T cell recruitment and activation, masking more subtle changes that might otherwise be detectable later in the dosing cycle.

## Resource availability

### Lead contact

Further information and requests for resources and reagents should be directed to and will be fulfilled by the lead contact, Peter Kirk (peter.kirk@immunocore.com).

### Materials availability

This study did not generate new unique reagents.

### Data and code availability

De-identified patient RNA-seq data have been deposited at EGA (European Genome-phenome Archive) as EGAD: 50000001258. They are available upon request if access is granted. To request access, go to https://ega-archive.org/and click on “Request access.” This paper does not report custom computer code. Any additional information required to reanalyze the data reported in this work is available from the lead contact upon request.

## Acknowledgments

Some results published here, as indicated, are based upon data generated by the TCGA Research Network: https://www.cancer.gov/tcga. The authors thank Revashnee Naidoo for her technical expertise and support in the lab, Alex Greenshield-Watson for data analysis and interpretation, and Anastasiya Kazachenka for BAP1 CNV analysis.

## Author contributions

Conception and design, Immunocore authors; provision of study material or patients, J.J.S., A.N.S., R.D.C., L.d.l.C.-M., Z.E., A.P.I., P.N., O.H., M.O.B., and T.S.; lab data generation, E.L., S.S., and C.B.-R.; bioinformatics, L.C. and S.K.; collection and assembly of data, Immunocore authors; data analysis and interpretation, all authors; manuscript writing, all authors; accountable for all aspects of the work, all authors.

## Declaration of interests

A.N.S. discloses grant/contract: Bristol Myers Squibb, Immunocore, Novartis, Targovax, Pfizer, Alkermes, Checkmate Pharmaceuticals, Foghorn Therapeutics, Linnaeus Therapeutics, Prelude Therapeutics, Iovance Biotherapeutics, Bristol Myers Squibb, Polaris, and Xcovery.

R.D.C. discloses consultant: Aura Biosciences, Castle Biosciences, Chimeron, Immunocore, InxMed, Iovance, Merck, OncoSec, Pierre Fabre Pharmaceuticals Inc., PureTech Health, Regeneron Pharmaceuticals, Rgenix, Sanofi Genzyme, Sorrento Therapeutics, and TriSalus; stock option: Aura Biosciences, Chimeron, and Rgenix.

A.P.I. discloses research funding to institution: Dynavax, GSK/Sarah Cannon, Immunocore, Merck, Neon Therapeutics/Sarah Cannon, and Checkmate Pharmaceuticals.

P.N. discloses data and safety monitoring: 4SC and Achilles; consultant/advisory board: 4SC, Bristol Myers Squibb, Immunocore, Merck, Merck Sharp and Dohme, Novartis, and Pfizer; research grant/contract: Immunocore.

O.H. discloses contract: Aduro Biotech, Akeso biotech, Amgen Inc., BeiGene Ltd, BioAtla, Bristol Myers Squibb, Genentech USA, Inc., GlaxoSmithKline, Idera Pharmaceuticals, Immunocore, Incyte Corporation, Janssen Global Services, LLC, Merck, Next Cure Inc., Novartis, Pfizer, Regeneron Pharmaceuticals Inc., Sanofi, Seattle Genetics, Tempus, and Zelluna Immunotherapy; contracted research for institution: Aduro Biotech, Akeso biotech, Amgen Inc., Arcus Biosciences, BioAtla, Bristol Myers Squibb, CytomX Therapeutics, Exelixis Inc., Genentech, GlaxoSmithKline, Idera Pharmaceuticals, Immunocore, Incyte Corporation, Iovance Biotherapeutics, Merck, Merck Serono, Moderna, NextCure Inc., Novartis, Pfizer, Regeneron Pharmaceuticals, Sanofi Genzyme, Seattle Genetics, Torque Pharma, and Zelluna Immunotherapy; speakers bureau: Bristol Myers Squibb, Novartis, and Pfizer.

M.O.B. discloses consultant/advisory: Adaptimmune, Bristol Myers Squibb Canada, GlaxoSmithKline, Immunocore, Instil Bio, Iovance Biotherapeutics, Merck, Novartis, Pfizer, Sanofi Pasteur Inc., Sun Pharma, IDEAYA Bio, Medison, Regeneron, and Iovance; safety review committee: GlaxoSmithKline and Adaptimmune; research funding: Merck, Takara Bio, and Novartis.

T.S. discloses advisory/consulting: Immunocore and Castle Biosciences; research funding to institution (clinical trials): Immunocore, Verastem, IDEAYA, TriSalus, and BMS.

L.d.l.C.-M. discloses Consultant/Advisory: MSD-Merck, Bristol Myers Squibb, Pierre-Fabré, Novartis, Gilead, Incyte, Daichii Sankyo, and AstraZeneca; stock ownership: none; research funding: MSD-Merck, Roche Farma, and Celgene; speaking: MSD-Merck, Roche Farma, Bristol Myers Squibb, Amgen, and Gilead; grant support: Bristol Myers Squibb, Roche Farma, and Gilead.

J.J.S. discloses PI on clinical trial: Amgen, AstraZeneca, Bristol Myers Squibb, Delcath Systems, Merck, Replimune, and Transgene; research grant/contract: AstraZeneca, Bristol Myers Squibb, and Immunocore; consultant/advisory board: Bristol Myers Squibb, Delcath Systems, Immunocore, Merck, and Replimune; congress attendance: Bristol Myers Squibb, Merck, and Replimune.

P.K., E.L., S.K., C.B.-R., L.C., S.S., and K.R. disclose employees and stock owners of Immunocore Ltd.

## STAR★Methods

### Key resources table

**Table:** 

REAGENT or RESOURCE	SOURCE	IDENTIFIER
**Antibodies**		

Gp100 (clone HMB45)	Roche Diagnostics	05479282001
CD3 (clone 2GV6)	Roche Diagnostics	05278422001; RRID: AB_2335978
CD8 (clone SP57)	Roche Diagnostics	05937248001; RRID: AB_2335985
CD20 (clone L26)	Roche Diagnostics	05267099001
Melanoma Triple Cocktail (clones HMB45 + A103 + T311)	Roche Diagnostics	06527787001
B2M (clone EPR21752-214)	Abcam	ab218230; RRID: AB_2943125
HLA-A (clone EP1395Y)	Abcam	ab52922; RRID: AB_881225

**Biological samples**		

Tumor biopsy samples	This study	N/A

**Critical commercial assays**		

TruSeq stranded mRNA library prep kit	Illumina	Cat# 20020595

**Deposited data**		

Raw RNASeq data	This paper	EGAD: 50000001258
Primary uveal melanoma gene expression data	https://www.cancer.gov/tcga	TCGA-UVM

**Software and algorithms**		

Trim Galore	Martin, M^64^	https://cutadapt.readthedocs.io/en/stable/
FastQC	Wingett SW, Andrews S	https://www.bioinformatics.babraham.ac.uk/projects/fastqc/
MultiQC	Ewels, P et al.^65^	https://github.com/MultiQC/MultiQC
STAR aligner	Dobin, A et al.^66^	https://github.com/alexdobin/STAR
RSEM	Li, B et al.^67^	https://github.com/deweylab/RSEM
R (version 4.4.1)	R Core Team	https://cran.r-project.org/
Burrows–Wheeler aligner	Li, H et al.^68^	https://github.com/lh3/bwa
Picard	Broad Institute	https://broadinstitute.github.io/picard/
Mutect2	Benjamin, D et al.^69^	https://github.com/broadinstitute/gatk/releases
GATK Somatic SNVs and INDELs	Van der Auwera, G.A. and O'Connor, B.D.^70^	https://github.com/broadinstitute/gatk/releases
PureCN package	Riester, M et al.^71^	https://bioconductor.org/packages/release/bioc/html/PureCN.html
Survminer package	Kassambara A	https://cran.r-project.org/web/packages/survminer/index.html
R Bioconductor	Gentleman R et al.	https://www.bioconductor.org/
ComplexHeatmap	Gu Z et al.	https://bioconductor.org/packages/release/bioc/html/ComplexHeatmap.html
DESeq2	Love, M.I et al.^72^	https://bioconductor.org/packages/release/bioc/html/DESeq2.html
clusterProfiler package	Yu G et al.^73^	https://bioconductor.org/packages/release/bioc/html/clusterProfiler.html
fgsea package	Korotkevich, G et al.^74^	https://bioconductor.org/packages/release/bioc/html/fgsea.html
Reactome PA	Yu G et al.^75^	https://bioconductor.org/packages/release/bioc/html/ReactomePA.html
HALO	Indica Labs	https://indicalab.com/halo/

### Experimental model and study participant details

Patient samples used in this study were collected as part of clinical trial NCT02570308 https://classic.clinicaltrials.gov/ct2/show/NCT02570308. This open-label, international, single-arm phase 1/2 study was composed of a phase 1 dose escalation and an initial expansion cohort that was subsequently expanded into a full phase 2 expansion study. The primary objective of the phase 1 portion of the study was to identify the maximum tolerated dose and determine the recommended phase 2 dose. The primary objective of the phase 2 portion was to estimate the objective response rate based on RECIST v1.1 in patients treated at the recommended phase 2 dose of tebentafusp. 146 patients were enrolled on the study. Age range at study enrollment was 25–88, median 61; gender was 51% female, 49% male; race was 99% white, 1% other. The trial was carried out in accordance with the principles of the Declaration of Helsinki and Good Clinical Practice guidelines, and the study protocol was approved by the relevant ethics bodies at each participating site: Princess Margaret Cancer Center, Toronto, Canada; Charite Universitaetsmedizin Berlin – Campus Benjamin Franklin, Berlin, Germany; Universitaetsklinikum Heidelberg, Heidelberg, Germany; Institut Catala d’Oncologia (ICO) l’Hospitalet, Hospital Duran i Reynals, Barcelona, Spain; Hospital Universitario Virgen Macarena, Seville, Spain; Centro de Investigación Biomédica en Red de Cáncer (CIBERONC), Madrid, Spain/Hospital Universitario La Paz, Madrid, Spain; Hospital General Universitario de Valencia, Valencia, Spain; The Clatterbridge Cancer Center, Wirral, UK; Mount Vernon Cancer Center, Northwood, UK; Columbia University Medical Center, New York, USA; Washington University School of Medicine, St Louis, USA; Thomas Jefferson University Hospital, Philadelphia, USA; Vanderbilt University Medical Center, Nashville, USA; Memorial Sloan Kettering Cancer Center, New York, USA; University of Colorado Cancer Center, Aurora, USA; The Angeles Clinic and Research Institute, a Cedars-Sinai Affiliate, Los Angeles, USA; H. Lee Moffitt Cancer Center and Research Institute, Inc., Tampa, USA; University of California San Diego Moores Cancer Center, La Jolla, USA; California Pacific Medical Center, San Francisco, USA; Baylor Scott & White Health, Dallas, USA; Dean A. McGee Eye Institute, University of Oklahoma, Oklahoma City, USA; Georgetown University – Lombardi Comprehensive Cancer Center, Washington, USA; University of Miami Hospital Clinics/Sylvester Comprehensive Cancer Center, USA; The University of Chicago Medical Center, Chicago, USA; Roswell Park Cancer Institute, Buffalo, USA; and Providence Portland Medical Center, Portland, USA. Patients provided written informed consent before being screened for enrollment.

Further study participant details can be found in Carvajal et al.^29^

### Method details

#### Patient tumor biopsy immunohistochemistry

Tumor biopsies were collected prior to first tebentafusp infusion, approximately 24 h after the third tebentafusp dose, and at time of clinical progression (determined by RECIST v1.1). Progression biopsies were taken prior to infusion or after discontinuation of treatment. Samples were processed by IHC, RNA sequencing and whole exome sequencing (baseline samples only). On-treatment (day 16) biopsies were optional in phase 1 and mandatory where medically feasible in phase 2. Only non-significant risk procedures were performed for accessing tumor tissue. All available biopsies passing QC criteria, such as adequate tumor content, were included in analysis.

Formalin fixed paraffin embedded tumor biopsy samples were sectioned and stained by IHC. Consecutive sections were used for H&E (to determine sufficient tumor content) and exploratory IHC markers. Only one slide was analyzed per single or dual stain.

For gp100 IHC; samples were stained with anti-melanosome (clone HMB45, Roche Diagnostics:05479282001) and UltraView Universal red and counterstained with haematoxylin on the Ventana Benchmark. Images were H-scored by a pathologist.

For all other IHC markers; samples were stained by single-plex IHC for CD3 or CD8 or by dual-plex IHC for B2M/CD3 or HLA-A/Melanoma Triple. The following primary antibodies were used: against CD3 (clone 2GV6, Roche Diagnostics:05278422001), CD8 (clone SP57, Roche Diagnostics:05937248001), CD20 (clone L26, Roche Diagnostics:05267099001), B2M (0.13μg/ml, clone EPR21752-214, Abcam:ab218230), HLA-A (0.5μg/ml, clone EP1395Y, Abcam:ab52922), Melanoma Triple Cocktail (clones HMB45 + A103 + T311,Roche Diagnostics:06527787001). Antibody staining was amplified using HQ-HRP, HRP or AP secondary antibodies and detected using the purple or yellow chromogen kits, slides were counterstained by hematoxylin and bluing reagents (Roche Diagnostics). Antibody staining was performed on the Roche Ventana autostainer. Slides were dehydrated and coverslipped. Images were acquired with the Pannoramic 250 FLASH III (3DHISTECH) Whole-Slide Scanner. Digital image analysis of the images was carried out using HALO software (Indica Labs). The number of positive CD3, CD8 or CD20 cells was quantified within the tumor and peri-tumoral stroma. Membrane specific HLA-A was quantified in tumor cells expressing the Melanoma triple marker. Lymphoid aggregates (LA) were identified using digital density analysis and defined as ≥10 CD20^+^ B cells within a CD3^+^ T cell aggregate of 100μm radius.

#### Patient tumor biopsy RNA sequencing

RNA sequencing libraries were generated from tumor biopsy samples, which had been placed in RNAlater or were snap frozen, using the Illumina TruSeq stranded mRNA kit at E.A.Genomics. Paired end fragments of 100 bp length were sequenced (50 million reads per sample) using the Illumina Novaseq system.

Following sequencing, raw FASTQ files were trimmed using Trim Galore (v0.6.2)^64^ and quality was assessed using FastQC and MultiQC (v1.9).^65^ The resulting reads were aligned using STAR aligner (v2.5).^66^ Reads were mapped to the GRCh38 primary assembly provided by Ensembl. Gene expression was quantified using RSEM (v 1.2.25).^67^ Transcript per kilobase per million (TPM) values were log2 transformed in R (version 4.4.1). A pseudo-count value of 1 was added to each TPM value prior to transformation.

The TCGA UVM dataset was obtained from the GDC data portal from the National Cancer Institute.

#### Tumor mutation analysis

Tumor biopsies were analyzed for mutations in *GNAQ*, *GNA11*, *SF3B1* and *BAP1* prior to tebentafusp treatment. DNA libraries were generated from tumor biopsy samples, which were snap frozen, using the Illumina ExomeSeq all exon v6 kit. Paired end fragments of 100 bp in length were sequenced (50 million reads per sample) using the Illumina NovaSeq system. The resulting reads were aligned using BWA-MEM (Burrows–Wheeler aligner – maximal exact match) v0.7.15.^68^ Reads were mapped to the GRCh38 primary assembly provided by Ensembl. Duplicate reads were flagged using the MarkDuplicate function of Picard to prevent variant call errors. Mutect2^69^ was run on process-matched normal samples to generate panel of normal database. Somatic variants were called using MuTect2 (GATK Somatic SNVs and INDELs 4.1.6.0).^70^

#### Copy number variant analysis

Copy number variation analysis was conducted using PureCN v2.6.3^71^ according to the workflow previously described by Oh et al.^76^ Interval coverage was calculated for process-matched healthy blood and tumor samples. Mutect2 was run on process-matched normal samples to generate panel of normal database. Tumor variants were called by Mutect2. Final copy number variations were called using PureCN normal coverage database, tumor Mutect2 filtered calls and tumor coverage files. 8q amplification was determined based on copy number at MYC and PTK2 loci which gave identical results.

### Quantification and statistical analysis

Sample grouping by CD3 IHC. Samples were classified by CD3 abundance into 3 groups using a threshold of 100 CD3 cells per mm^2^; low CD3 in tumor and stroma = deserted, low tumor and high stroma = excluded, high in tumor = Inflamed.

Survival analysis was carried out using the R package survminer v0.4.9; the Cox log rank was used to assess differences between the survival curves. Kaplain-Meier analysis of samples collected at the time of progression was landmarked to 90 days.

Fisher exact test was used to assess associations between patient groups, defined by baseline and on-treatment expression measurements, and clinically derived outcomes. The Wilcoxon rank-sum test was used to assess associations between baseline and on-treatment levels of expression. These tests were two sided and were carried out using R stats package 4.1.

Euclidean distance was applied per patient to generate a distance matrix and complete-linkage clustering was carried out. Heatmap generated using R Bioconductor (v3.16) package ComplexHeatmap (v.2.14.0). Whole transcriptome correlation analysis was carried out using the Pearson method.

Differential gene expression analysis on 35 paired patients between baseline and day 16 on-treatment was conducted using Bioconductor package DESeq2 (v1.38.3)^72^ with significant genes defined by p_adj_ < 0.05 and absolute log2FoldChange>0.75. Batch was accounted for within DESeq2, and to minimize batch variation effect paired samples from the same patient were analyzed within the same batch.

For gene ontology over-representation analysis, the clusterProfiler package (v4.10.0)^73^ and ReactomePA (v1.46)^75^ was used for reactome pathway over-representation analysis.

Differential gene expression in gp100 high and low groups was carried out between baseline and day 16 on-treatment using the Bioconductor package DESeq2 (v1.38.3).

For the APM gene signature the median gene expression of *HLA-A*, *B2M*, *TAP1*, *TAP2*, *PSMB8*, *PSMB9* and *TAPBP* was calculated per patient.

GSEA was carried out to compare baseline and on-treatment up-regulated genes using ReactomePA. GSEA of liver associated genes was assessed using fgsea (v1.3)^74^ using the human SU_LIVER Molecular Signatures Database gene set.^77^

### Additional resources

Patient samples used in this study were collected as part of clinical trial NCT02570308.

https://classic.clinicaltrials.gov/ct2/show/NCT02570308.

## Supplemental information

Document S1. Figures S1–S8 and Tables S1–S3

Table S4. Anatomical site of biopsies analyzed by RNA-seq, related to Table 1 and Figure 2

Table S5. Gene expression at baseline, related to Figure 2

Table S6. Gene expression on-treatment, related to Figure 4

Table S7. Gene expression at progression, related to Figure 6

Table S8. Patient metadata, related to Figures 1 and 2 and Table 1

Document S2. Article plus supplemental information
