# Supplementary material for: Evolution of the tumor immune landscape during treatment with tebentafusp, a T cell receptor-CD3 bispecific
Source: Cell Rep Med. 2025 Apr 15;6(4):102076. doi: 10.1016/j.xcrm.2025.102076 (PMC12047528; doi:10.1016/j.xcrm.2025.102076)
Supplement: Document S1. Figures S1–S8 and Tables S1–S3 [file mmc1.pdf]

Cell Reports Medicine, Volume *6*

# Supplemental information

# Evolution of the tumor immune landscape during treatment with tebentafusp, a T cell receptor-CD3 bispecific

**Joseph J. Sacco, Peter Kirk, Emma Leach, Alexander N. Shoushtari, Richard D. Carvajal, Camille Britton-Rivet, Sophie Khakoo, Laura Collins, Luis de la Cruz-Merino, Zeynep Eroglu, Alexandra P. Ikeguchi, Paul Nathan, Omid Hamid, Marcus O. Butler, Sarah Stanhope, Koustubh Ranade, and Takami Sato**

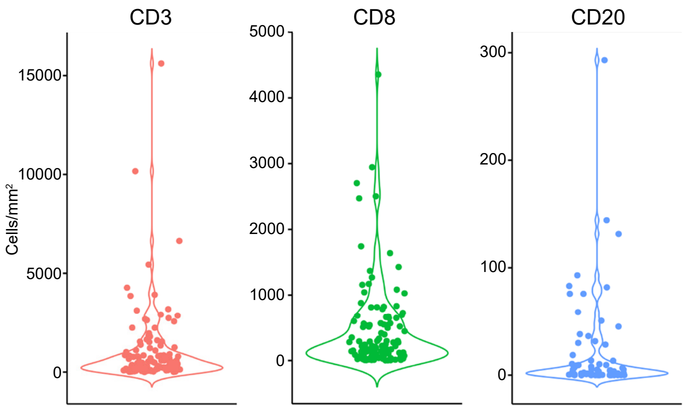


**Figure S1. Distribution of abundance of CD3, CD8, and CD20-positive cells in baseline tumor biopsies.** Baseline tumor biopsies were stained for CD3, CD8 and CD20 by IHC, and marker-positive cells within tumor regions enumerated. Related to Figure 1.

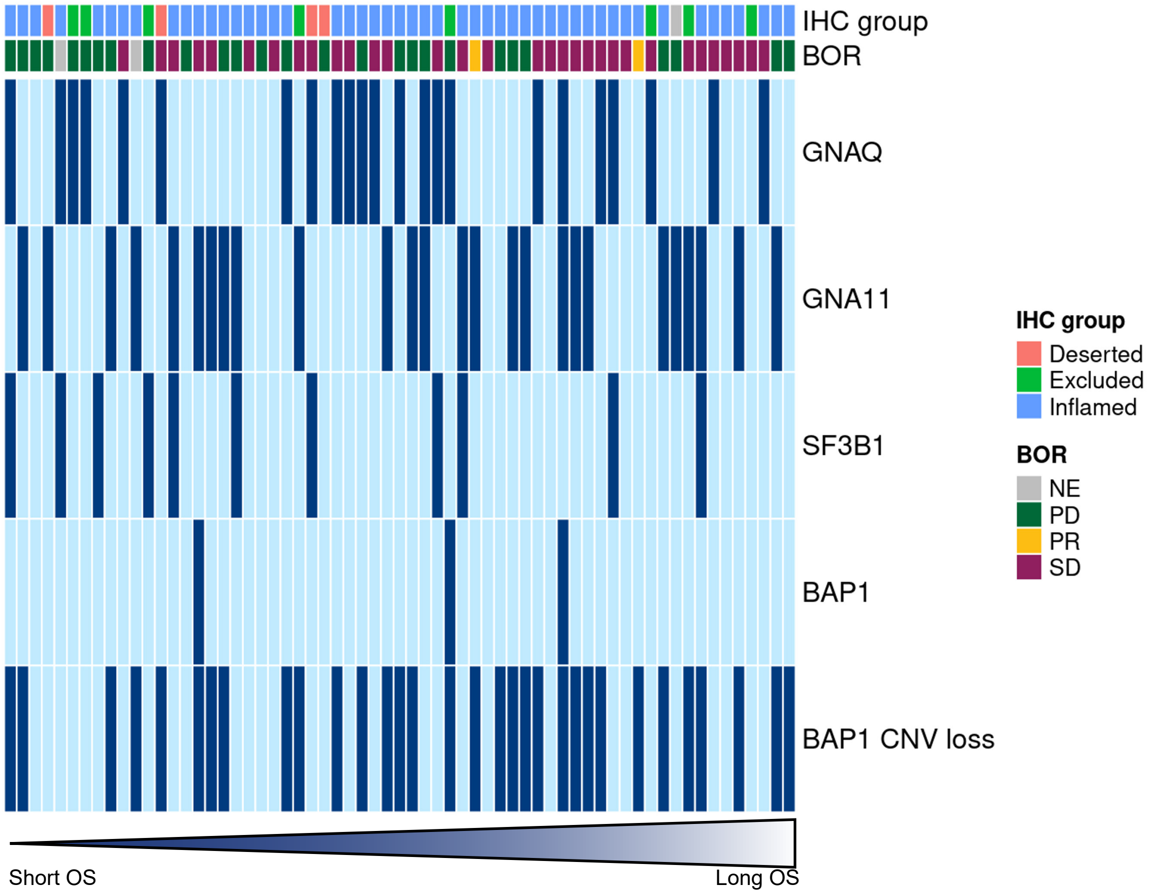


**Figure S2. Oncoprint showing distribution of selected genomic alterations** based on whole exome sequencing of baseline tumor biopsies. Dark blue indicates presence of mutation or (for BAP1 only) copy number variation. Related to Figure 1 and Table 1.

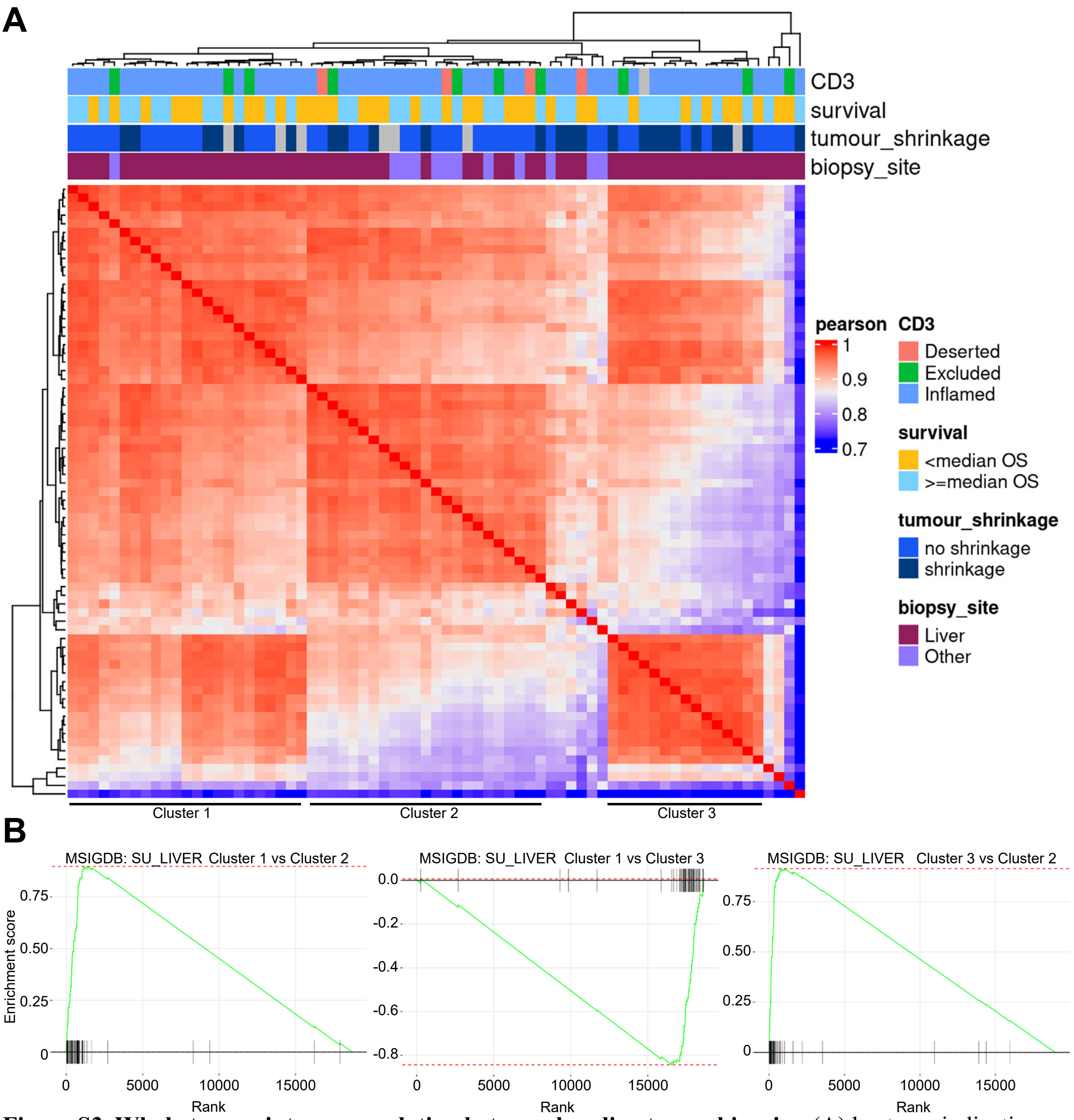


**Figure S3. Whole transcriptome correlation between baseline tumor biopsies.** (A) heatmap indicating correlation (Pearson coefficient) between whole transcriptomes of baseline biopsies, with ribbons indicating CD3+ cell infiltration status (see Figure 1D), overall survival, tumor shrinkage and biopsy site (B) GSEA analysis of differential expression of liver genes (MSIGDB:SU_LIVER) between the three major clusters. Related to Figure 2.

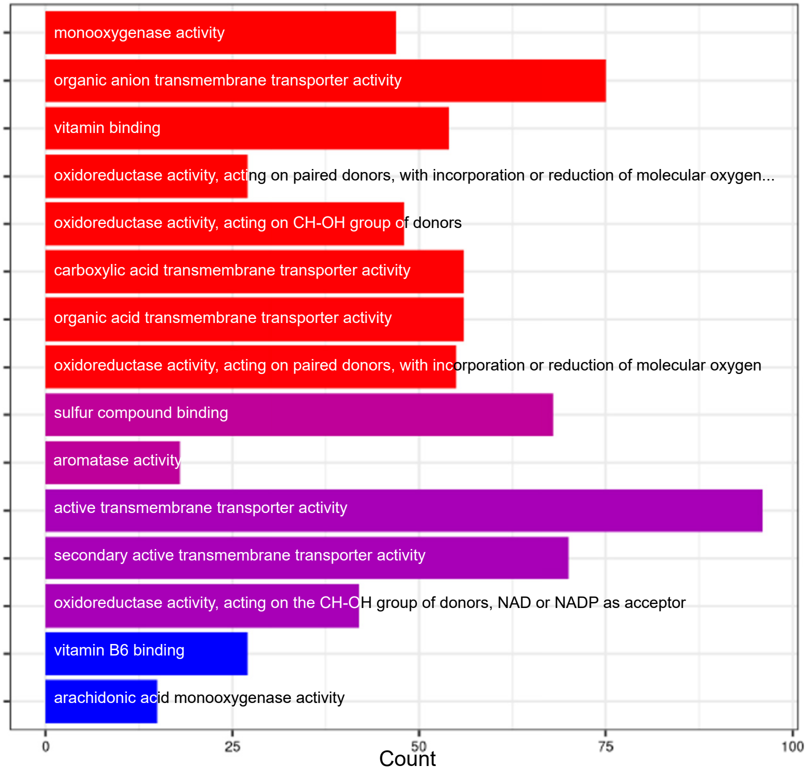


**Figure S4. Comparison of gene expression in baseline tumor biopsies from liver metastases vs from other sites.** Pathway analysis was performed on differential gen expression based on site of biopsy (liver vs non-liver). Pathways with the most significantly elevated expression in samples from liver are shown. Related to Figure 2.

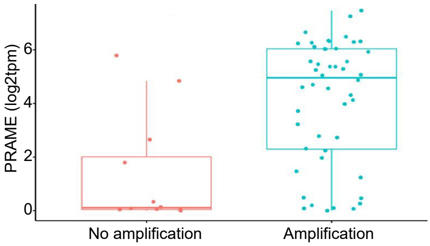


**Figure S5. PRAME gene expression in tumor biopsies, by 8q amplification status.** Boxes indicate median and interquartile range. Median expression 29-fold higher in patients with 8q amplification, $p<0.0005$. Related to Figure 2.

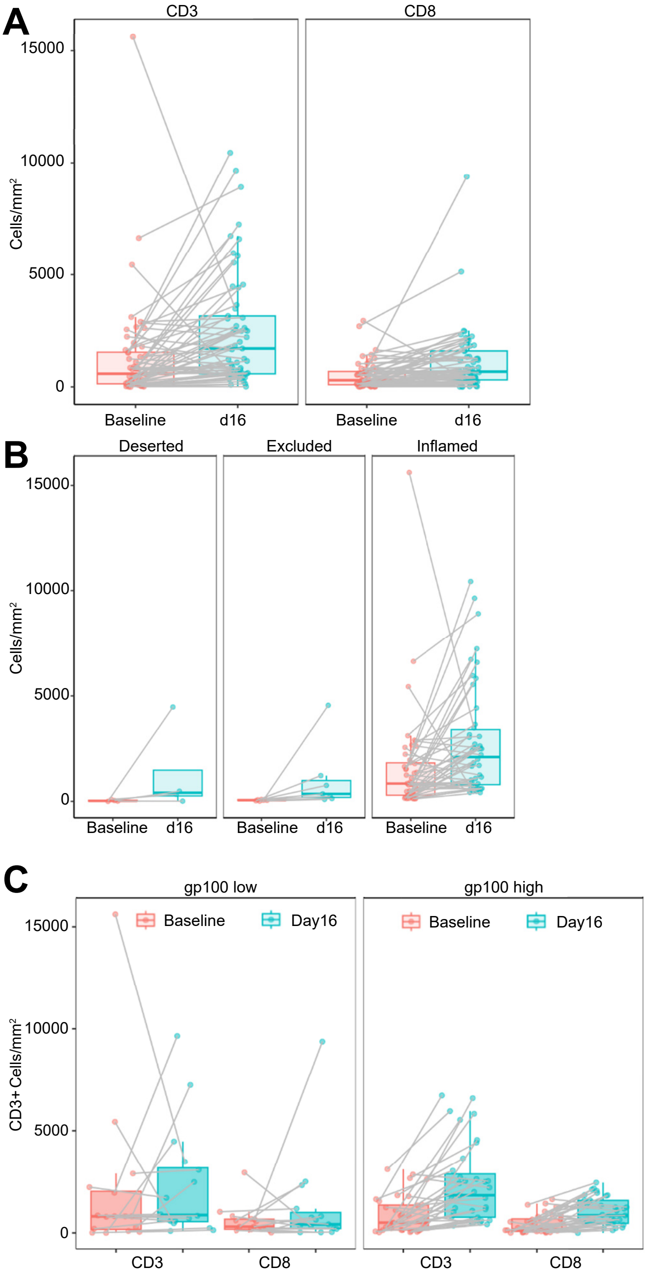


**Figure S6. Change in abundance of CD3 and CD8 T cells in paired biopsies.** Data is identical to figures (A) 3A, (B) 3B and (C) 5A, with the addition of lines joining paired biopsies from the same patient. Median and inter-quartile range are indicated. Related to Figure 3 and Figure 5.

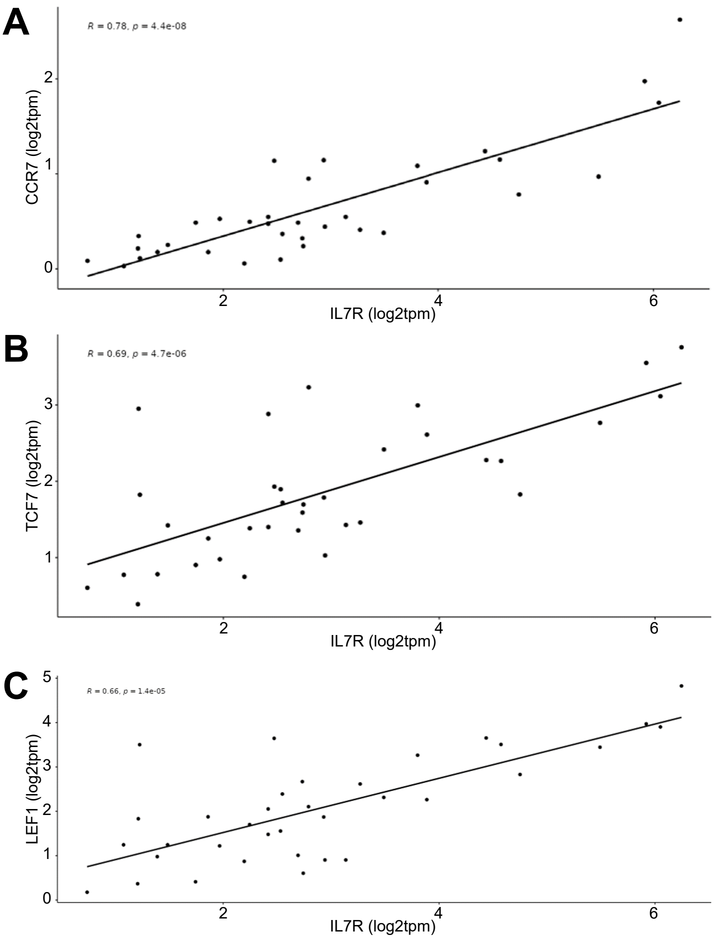


**Figure S7. Correlation of IL7R expression in tumor at d16 with genes associated with naive subset and stemness in T cells.** (A) CCR7 (R=0.78, p=4.4R-8), (B) TCF7 (R=0.69. p=4.7E-6), (C) LEF1 (R=0.66, p=1.4E-5). Related to Figure 4.

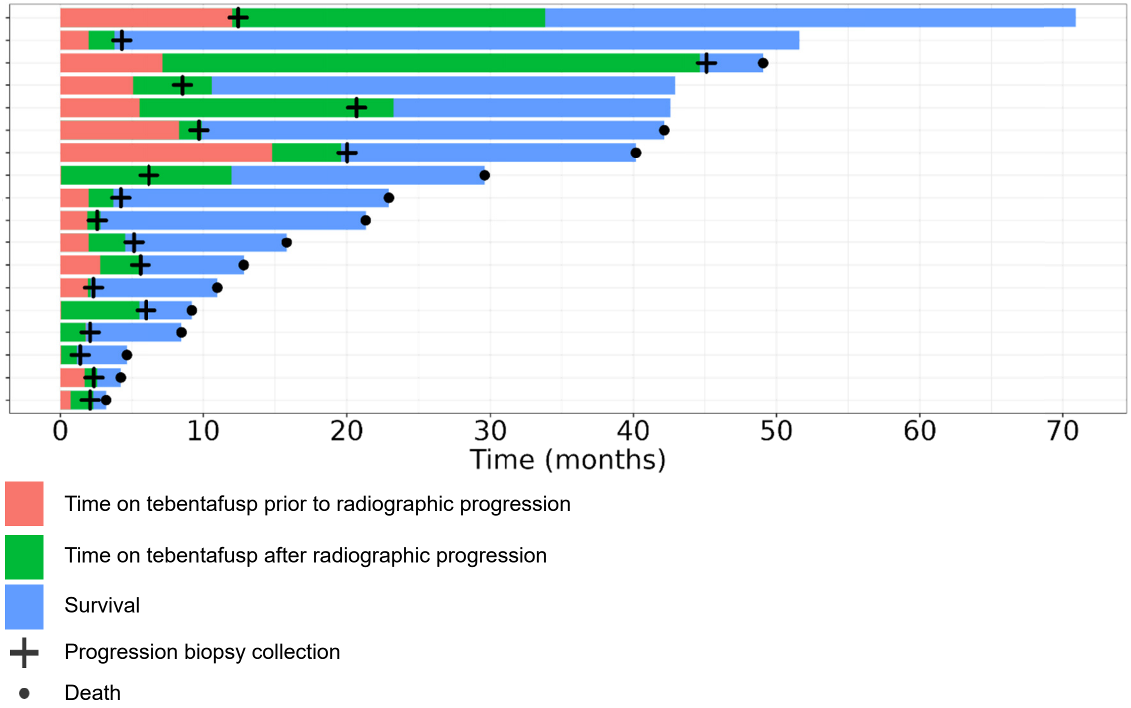


**Figure S8. Timing of progression biopsies.** Swim-lane plot indicates time of radiographic progression, progression biopsy, and survival beyond progression for patients from whom progression biopsies were collected. Related to Figure 6.

## Supplemental Tables S1 – S3

**Table S1 – anatomical sites of biopsy** Related to Table 1, Figures 1-7.

| **IHC** | Baseline | D16 | Progression |
|---|---|---|---|
| Liver | 115 | 54 | 14 |
| Lymph node | 3 | 0 | 0 |
| Soft tissue | 2 | 0 | 1 |
| Eye | 2 | 0 | 0 |
| Other | 24 | 14 | 2 |
| Unknown | 1 | 1 | 1 |

| **RNASeq** | Baseline | D16 | Progression |
|---|---|---|---|
| Liver | 59 | 39 | 12 |
| Lymph node | 0 | 0 | 0 |
| Soft tissue | 1 | 0 | 0 |
| Other | 11 | 10 | 2 |
| Unknown | 0 | 0 | 0 |

**Table S2 – Genes associated at baseline with both overall survival (OS) and tumor reduction (TR)** Related to Figure 2.

| Gene | OS Hazard Ratio | OS p-value | TR Odds Ratio | TR p-value |
|---|---|---|---|---|
| UBA7 | 0.313 | <0.001 | 0.13 | 0.001 |
| WDR86 | 0.436 | 0.001 | 0.1 | <0.001 |
| ANKRD49 | 0.503 | 0.008 | 0.09 | <0.001 |
| TMC8 | 0.417 | 0.001 | 0.13 | 0.001 |
| IGLV1-51 | 0.439 | 0.001 | 0.13 | 0.001 |
| NR3C1 | 0.323 | <0.001 | 0.18 | 0.004 |
| FOXD4L1 | 0.494 | 0.006 | 0.12 | 0.001 |
| ZNF846 | 0.481 | 0.005 | 0.13 | 0.001 |
| RIMBP2 | 0.422 | 0.001 | 0.15 | 0.001 |
| GRAPL | 0.504 | 0.008 | 0.13 | 0.001 |
| IGHD | 0.465 | 0.003 | 0.15 | 0.001 |
| ITSN2 | 0.485 | 0.005 | 0.15 | 0.001 |
| IGHG2 | 0.408 | 0.001 | 0.18 | 0.004 |
| CLIC3 | 0.442 | 0.001 | 0.18 | 0.004 |
| LGR6 | 0.443 | 0.003 | 0.18 | 0.004 |
| STAT4 | 0.45 | 0.002 | 0.18 | 0.004 |
| TSPO2 | 0.511 | 0.008 | 0.16 | 0.002 |
| RHEX | 0.457 | 0.003 | 0.18 | 0.004 |
| LRP1B | 0.401 | <0.001 | 0.21 | 0.005 |
| RHOXF2 | 0.468 | 0.004 | 0.18 | 0.004 |
| FOXP1 | 0.471 | 0.004 | 0.18 | 0.004 |
| LDLRAD2 | 0.472 | 0.003 | 0.18 | 0.004 |
| PCNX1 | 0.493 | 0.006 | 0.18 | 0.004 |
| CAMK1D | 0.494 | 0.007 | 0.18 | 0.004 |
| PLEKHM3 | 0.499 | 0.009 | 0.18 | 0.004 |
| TENT5A | 0.499 | 0.008 | 0.18 | 0.004 |
| REPS1 | 0.5 | 0.007 | 0.18 | 0.004 |
| TNNT2 | 0.504 | 0.007 | 0.18 | 0.004 |
| KIAA0408 | 0.506 | 0.007 | 0.18 | 0.004 |
| PTK2B | 0.506 | 0.009 | 0.18 | 0.004 |
| TMSB4XP2 | 0.51 | 0.008 | 0.18 | 0.004 |
| TSC22D3 | 0.512 | 0.009 | 0.18 | 0.004 |
| CHMP5P1 | 0.519 | 0.01 | 0.18 | 0.004 |
| DGKB | 0.426 | 0.001 | 0.23 | 0.01 |
| ADGRG2 | 0.476 | 0.004 | 0.21 | 0.005 |
| PHF10P1 | 0.481 | 0.004 | 0.23 | 0.01 |
| PRDM16 | 0.511 | 0.009 | 0.23 | 0.01 |
| PDCL3P2 | 0.524 | 0.01 | 0.23 | 0.01 |

**Table S3 – Fold-change in median gene expression** between pre-treatment and d16 biopsies, and associated unadjusted p-value, for selected genes referenced in text. Related to Figure 4.

| **Gene** | **Fold-change** | **p-value** |
|---|---|---|
| CXCL9 | 8.69 | 0.000018 |
| FDCSP | 5.1 | 0.000055 |
| GBP1 | 4.53 | 0.0000078 |
| IDO1 | 4.51 | 0.000015 |
| GBP2 | 4.12 | 0.000010 |
| CXCL11 | 3.72 | 0.00024 |
| IFI44L | 3.27 | 0.000022 |
| ETV7 | 2.22 | 0.00013 |
| HAVCR2 | 2.2 | 0.0084 |
| MLKL | 2.04 | 0.00025 |
| UBA7 | 2.04 | 0.00010 |
| CASP1 | 2.03 | 0.00048 |
| CD274 | 2.02 | 0.00027 |
| GSDMD | 1.84 | 0.00059 |
| CTLA4 | 1.73 | 0.0011 |
| GSDMB | 1.71 | 0.010 |
| RIPK3 | 1.63 | 0.00094 |
| NLRP3 | 1.49 | 0.0026 |
| PYCARD | 1.45 | 0.042 |
| LAG3 | 1.32 | 0.017 |
| KLRB1 | 1.14 | 0.16 |
| FOXP3 | 1.08 | 0.37 |
| NOS2 | 0.99 | 0.72 |
| KLRF1 | 0.97 | 0.51 |
| ARG1 | 0.36 | 0.23 |
